# Initial evidence for neural correlates following a therapeutic intervention: altered resting state functional connectivity in the default mode network following attention training technique

**DOI:** 10.3389/fpsyt.2025.1479283

**Published:** 2025-03-06

**Authors:** Torben Müller, Svenja Krug, Özlem Kayali, Erik Leichter, Niklas Jahn, Lotta Winter, Tillmann H. C. Krüger, Kai G. Kahl, Christopher Sinke, Ivo Heitland

**Affiliations:** ^1^ Department of Psychiatry, Social Psychiatry and Psychotherapy, Hannover Medical School, Hanover, Germany; ^2^ Division of Clinical Psychology and Sexual Medicine, Department of Psychiatry, Social Psychiatry and Psychotherapy, Hannover Medical School, Hanover, Germany; ^3^ Center for Systems Neuroscience Hannover, Hanover, Germany

**Keywords:** attention training technique, major depressive disorder, default mode network, resting-state functional connectivity, posterior cingulate cortex, middle frontal gyrus

## Abstract

**Introduction:**

The Attention Training Technique (ATT) is a psychotherapeutic intervention in Metacogntive Therapy (MCT) and aims at reducing maladaptive processes by strengthening attentional flexibility. ATT has demonstrated efficacy in treating depression on a clinical level. Here, we evaluated ATT at the neural level. We examined functional connectivity (FC) of the default mode network (DMN).

**Method:**

48 individuals diagnosed with Major Depressive Disorder (MDD) and 51 healthy controls (HC) participated in a resting-state (rs) *functional magnetic resonance imaging* (fMRI) experiment. The participants received either one week of ATT or a sham intervention. Rs-fMRI scans before and after treatment were compared using seed-to-voxel analysis.

**Results:**

The 2x2x2 analysis did not reach significance. Nevertheless, a resting-state connectivity effect was found on the basis of a posttest at the second measurement time point in MDD. After one week, MDD patients who had received ATT intervention presented lower functional connectivity between the left posterior cingulate cortex (PCC) and the bilateral middle frontal gyrus (MFG) as well as between the right PCC and the left MFG compared to the MDD patients in the sham group. In HC we observed higher rsFC in spatially close but not the same brain regions under the same experimental condition.

**Conclusion:**

We found a first hint of a change at the neural level on the basis of ATT. Whether the changes in rsFC found here indicate an improvement in the flexible shift of attentional focus due to ATT needs to be investigated in further research paradigms. Further experiments have to show whether this change in functional connectivity can be used as a specific outcome measure of ATT treatment.

## 1 Introduction

Major depressive disorder (MDD) is one of the most common psychiatric disorders. It is affecting over 322 million people worldwide every year and its core symptoms are depressed mood or loss of interest (1). The clinical presentation of depression is also very heterogeneous, which presumably also contributes to the high diversity of theoretical explanatory approaches and the large number of results on the psychopathology of MDD. For example, patients with very different combinations of diagnostic criteria can fulfil the diagnosis of MDD (2–4). Medication or psychotherapy can be used to treat MDD (5). In the context of psychotherapy, several interesting therapy methods with various interventions have been developed in recent decades (e.g. behavioral activation, cognitive behavioral therapy, acceptance and commitment therapy). Another method is Metacognitive Therapy (MCT), which we focus on here. This therapy is based on the self-regulatory executive function model (S-REF; 6) model, which has expanded the understanding of metacognitions in the genesis of mental disorders (7, 8) and provides a cognitive model for a specific group of symptoms represented by CAS. Central to the S-REF is the Cognitive Attentional deficit Syndrome (CAS), which involves the ongoing use of maladaptive self-regulatory strategies, such as rumination, and consequently exacerbates emotional distress (8). CAS is characterized by inflexible attentional control and encompasses several psychologically maladaptive cognitive processes such as rumination, worry or threat monitoring (9). Ruminating as a maladaptive cognitive strategy binds attentional resources and thus maintains the disorder (10). An important tool in the application of Metacognitive Therapy (MCT) is the Attention Training Technique (ATT), which aims to disengage attention from the use of CAS strategies through specific exercises. The Attention Training Technique (ATT) is a 12-minute intervention that should accompany psychotherapy in MCT and aims to reduce CAS by strengthening attentional flexibility and control (11). A combination of three attention exercises (selective attention, rapid attention switch, divided attention; 12) enables the individual to better shift attention away from maladaptive processes and thus interrupt CAS (9). ATT can be performed as a standalone treatment. The effect of ATT on improving attentional flexibility is well established in clinical samples at the behavioral level (for a review see 9). However, to date, only four studies investigate the neural correlates of ATT using neuropsychological methods. One study included only six individuals, resulting in low statistical power (13). One other study used only a single dose intervention of ATT, which is not compliant with the MCT manual (14). Two studies used imaging methods different from those used here. Knowles and Wells (15) used electroencephalography EEG instead of fMRI and Rosenbaum et al. (16) used functional near-infrared spectroscopy (fNRS). Based on the theoretical assumptions of MCT and the effects of ATT observed so far, it can be concluded that ATT could probably contribute to an improvement in the voluntary flexible shifting of attentional focus (17). If so, then ATT treatment at the neural level should be detectable in the areas associated with functions of self-referential processing (MFG; 18) and shifting attention (PCC; 19). ATT has demonstrated efficacy in treating depression on a clinical level (9, 14). In psychotherapeutic practice, ATT should be applied repeatedly during therapy according to the manual (11). To increase external validity, the intervention should therefore be conducted with several doses. No studies have yet examined the effects of a high-dose ATT intervention at the neurobiological level using fMRI. If the characteristics of CAS (e.g. rumination) are associated with dysregulation of the medial areas of the DMN (20) and ATT decreases rumination at the behavioral level, it is reasonable to hypothesize that ATT should be accompanied by a demonstrable reduction of functional connectivity in the core regions of the DMN. In the context of the normalization hypothesis, the rsFC in the DMN of patients with MDD should be downregulated to the level of HC after the application of ATT.

On a neural level, patients show impairments in brain circuits involved in cognitive (21) and emotional processing (22). There are different explanatory models for MDD focusing on different regions (e.g. insula, ACC, PCC, hippocampus). One interesting line of research that we would like to refer to deals with the DMN, which proposed MDD as a network-based disorder (23, 24). Accordingly, imaging studies have shown reliable changes in resting-state (rs) functional connectivity (FC) of network-connected areas (25, 26). Interestingly, studies often only found components of these networks. Therefore, until now, the results of these studies on the neural correlates of MDD vary considerably (27) and thus do not yet provide a coherent explanatory model for the cognitive and emotional pathologies of MDD (28). Despite various theoretical approaches and efforts to optimize treatment (26, 29), the etiology and pathophysiology of MDD remain controversial. This is likely related to the absence of large datasets and highly heterogenic samples, leading to different results in empirical studies (20, 27, 30).

Sheline et al. (31) had already proposed an MDD model in which higher activation of the Default Mode Network (DMN) plays a central role (32). This assumption is supported by neuroimaging studies that found systematically higher rsFC between certain brain areas of the DMN using functional magnetic resonance imaging (fMRI) (29, 30). The DMN is active when the brain is not engaged in an external task (33) and higher rsFC within the DMN is thought to be involved in self-related processes (26), negative rumination processes (34), deficits in autobiographical memory (35), problem solving and attentional processes (36). The DMN is often differentiated into a midline core system with major nodes in the posterior cingulate cortex (PCC) and medial prefrontal cortex (mPFC) and a subsystem with a major node in the medial temporal lobe (mTL) (33). The midline system is often associated with self-referential affective decision-making and the temporal lobe system with future-oriented thinking based on the retrieval of autobiographical memories (37, 38). The DMN is a promising candidate for testing the neural basis of psychotherapeutic interventions (24) and may contribute to a better understanding of MDD. Furthermore, it is hypothesized that the individual DMN connectivity pattern can be used for choosing the right treatment and thus increase the effectiveness of interventions (39). For example, studies were able to distinguish individuals with a high familial risk of developing MDD from individuals at low familial risk based on their levels of rsFC in the DMN (40). In addition, the connectivity patterns of various brain areas within the DMN were used to identify individuals with and without MDD with high accuracy (41). Moreover, rsFC in the DMN can be applied to distinguish different subtypes of depression (36).

Besides the DMN, also other brain networks have been associated with MDD, often reported are alterations in the salience network (SN). This network comprises the insula, the anterior cingulate cortex (ACC), the amygdala and the temporal pole and is activated when prominent salient stimuli occur, but also in stressful situations. Moreover, it is presumed to play a key role in paralimbic emotional processing and emotional control (24). The executive control network (EXE) is involved in cognitive processing, such as working memory, decision making, and attentional circuitry (25). Containing the dorsolateral prefrontal and parietal areas (42) and overlaps with regions of the DMN (43), it is thought to be involved in top-down modulation of attentional and working memory tasks such as regulating attention to internal or external stimuli (44, 45). In the SN and EXE, the direction of significant alterations remains inconsistent (23, 26). The cortico-limbic network (LIM) comprises the hippocampus, the amygdala and the ACC. Alteration (hyper- and hypoconnectivity) of the hippocampus to other regions were reported in MDD. The hippocampus plays an essential role in memory and cognitive functions (41, 46).

Resting-state connectivity patterns can also differentiate different psychiatric disorders in the human brain (47) or indicate the efficacy of psychotropic drugs (48) or psychotherapeutic interventions (36). At present, despite drug medication psychotherapy is a treatment of choice in most depressed individuals, with or without combined psychopharmacological treatment (5, 36), but do not lead to recovery in every patient (49). To date, there is no consensus on the areas of the brain that can predict or demonstrate response to psychotherapeutic interventions in MDD (50), as already the affected areas of the DMN and the direction of change between these areas (e.g. higher or lower rs-FC) vary greatly between studies (24, 26). For MDD, the majority of studies found hyperactivation in the DMN (e.g. 33, 51), but some studies also found reduced connectivity (29). Since MDD is characterized by significantly increased connectivity in the DMN, it has been hypothesized that psychotherapy at the neural level might be associated with a normalization of this connectivity (52).

Studies that investigated the influence of psychotherapeutic interventions on brain networks using fMRI (rsFC) found the following results. Studies have shown alterations in the rsFC of the DMN of patients with MDD during various therapeutic interventions: Behavioral activation (53), emotion regulation therapy (19) and group psychotherapy (54). The majority of studies focused on cognitive behavioral therapy (CBT; for an overview see 55). To our knowledge, there is not enough research on neural models for Metacognitive Therapy (MCT) yet. However, studies on therapy methods similar to MCT have already shown good clinical effects (10). MCT is a well-suited candidate for investigation, because it provides an expansion of the understanding of the development and maintenance of psychiatric disorders such as MDD (7, 56, 57).

## 2 Materials and methods

### 2.1 Participants

48 participants with major depression disorder (MDD) were recruited from the department of Psychiatry, Social Psychiatry and Psychotherapy of the Hannover Medical School and via advertisements in local psychotherapists’ offices. Additionally, 51 healthy controls were recruited via advertisement on the intranet of Hannover Medical School. All participants reported being right-handed and free from neurological conditions and episodes of alcohol or drug abuse for at least three months. Healthy subjects and MDD diagnosis were confirmed by a structured clinical interview for DSM-IV (SCID; 58) conducted by trained interviewers/psychologists of the Medical School Hannover. The current study contains a sample of individuals that participated in studies performed in our lab (59, 60) and was designed using insights of previous studies in our lab (61, 62).

All study procedures were performed in accordance with the Declaration of Helsinki (63) and were approved by the local ethics committee of the Hannover Medical School. Participants gave written and informed consent prior to participation and received financial compensation. Participants with depression were assigned into two well-matched sample groups showing no significant differences in sex, age or BDI-II-score. First group comprised 18 female and 7 male individuals, mean age 32.72 ± 10.93, mean BDI-II 29.36 ± 9.29. Second group comprised 12 female and 11 male participants, mean age 37.57 ± 11.6, mean BDI-II 31.39 ± 11.4 (**Table 1**).

**Table 1:** Demographic and clinical data of patients with MDD and HC who underwent ATT or Sham.

	ATT	Sham	t value	p value
MDD				
N	25	23		
Biological sex (F/M)	18/7	12/11	2.009	0.156^a^
Age (in years, mean, SD)	32.72 ± 10.93	37.57 ± 11.06	-1.146	0.252^b^
T1_BDI-II (mean score, SD)	29.36 ± 9.29	31.39 ± 11.4	-1.167	0.243^b^
T2_BDI-II (mean score, SD)	26.83 ± 12.44	30.43 ± 11.22	-1.012	0.317^c^
HC				
N	25	26		
Biological sex (F/M)	16/9	16/10	0.033	0.856^a^
Age (in years, mean, SD)	33.36 ± 8.35	34.08 ± 7.32	-0.652	0.514^b^
T1_BDI-II (mean score, SD)	4.5 ± 6.20	5.04 ± 6.61	-0.248	0.804^b^
T2_BDI-II (mean score, SD)	2.83 ± 5.10	4.00 ± 7.15	-0.110	0.912^b^

Table shows means and standard deviations of the demographic and clinical features of patients with Major Depressive Disorder (MDD) and healthy controls (HC). F, female; M, male; SD, Standard deviation; BDI-II, Beck Depression Inventory; a, Pearson-Chi-Square; b, Mann-Whitney-U Test; c, student’s t-test.

### 2.2 Procedure

Resting-state MRI data was acquired in the context of a larger fMRI study in our department in which Attention Training Technique (ATT) as a treatment in the framework of metacognitive therapy was examined. The results presented here are part of a group of studies containing a sample of healthy individuals that participated in a study performed in our lab (59). After recruiting 48 participants with major depression disorder, we were able to compare both groups. In a previous paper we compared both groups at baseline to gain insides about alterations of resting state networks in major depression (Krug et al., 2022). The study was conducted as a randomized, double-blind, placebo-controlled study using an online randomizer. Within these both groups the participant were randomly allocated to conceive either ATT or sham training as the only difference in the tests. Participants were required to appear at the study site on two different dates. An interval of one week was chosen, in which participants were assigned either ATT or sham training (see the ATT paragraph below for more information). Both groups were asked to practice ATT or sham daily at home. The training time each day was 23 minutes in total. Participants were asked to record their training times as well as any omissions, incomplete trainings or sudden interruptions.

### 2.3 The attention training technique

A standardized audio file in German language was used according to the ATT instructions/MCT manual (11). Each participant received a total of 16 sessions of ATT (2 sessions (23 minutes) per day for 8 days). On day 1 (at the end of the first experimental day) and day 8 (at the beginning of the second experimental session) participants listened to the audio tape on site. Between day 2 and day 7, individuals were asked to train every day at home. One session with the ATT audio file includes three different stages: selective attention, attentional switching, and divided attention. The ATT tape begins with instructions, which tell the participant which sounds to focus on. Six audio tracks are played simultaneously: crickets chirping, traffic noise, a tolling bell, rushing water, a ticking clock and twittering birds. The German versions of the ATT/sham training, which were used in this study, can be obtained at http://www.metakognitivetherapie.de.

### 2.4 Sham training

Participants receiving sham training followed the same procedure, but the instructions on the audio tape were missing. The participants did not receive any information regarding the three phases of selective attention, attentional switching, and divided attention, but instead just listened to the six overlapping audio tracks.

### 2.5 Neuroimaging

MRI images were acquired using a 3.0-T Siemens MAGNETOM Skyra scanner running Syngo VE11 with a 64-channel head coil. Acquisition of the 470 volumes of the rs-data lasts 10min 21s using a gradient simultaneous multislice EPI T2* sensitive sequences (78 slices, voxel size = 2 X 2 X 2 mm, TR = 1,31s, TE = 36ms, FOV = 208 X 208mm, flip angle = 64°, acceleration factor 6). During this scan, participants were asked to keep their eyes open and look at a white cross on black background on a 32inch Neuro-Nordic-Lab (NNL) monitor via a mirror. Afterwards, an individual high resolution anatomical image was acquired for each participant using a T1 weighted magnetization prepared rapid acquisition gradient echo sequence (208 slices, resolution = 1 X 1 X 1mm, TR = 2,4s, TE = 2.13ms, FOV = 192 X 246mm, flip angle = 8°).

### 2.6 fMRI pre-processing

The Matlab based software Statistical Parametric Mapping (SPM) version 12 (Wellcome Department of Imaging Neuroscience, University College London, UK) and DPABI 4.0 (64, 65) were used for data analysis. The following data processing steps were performed according to the standard protocol as described by Song et al (64) and Yan et al. (65). The first five images were removed to account for instability of the initial signal and the adaptation of the participants to the scanner. Images were slice time corrected and realigned to a mean image and then spatially normalized to the Neurological Institute (MNI) stereotaxic space using unified segmentation on T1 image (66) and resampled to 2 × 2 × 2 mm^3^. All undesired signals regressed out (67): Voxel-specific 12 motion parameters (67, 68), white matter signal, cerebrospinal fluid signal, and global signal was regressed out, as this last processing step contributes to improve the specificity of FC (69) and can improve the correction of motion artifacts (68). Images were then band-pass filtered (0.01–0.1 Hz). Afterwards, motion scrubbing procedure was applied by removing scans with a frame-wise displacement (FWD) threshold of > 0.4 mm, as described by Jenkinson (70). Finally, all images were smoothed using a 4x4x4mm FWHM Gaussian kernel.

### 2.7 ROI selection

**Figure 1:**
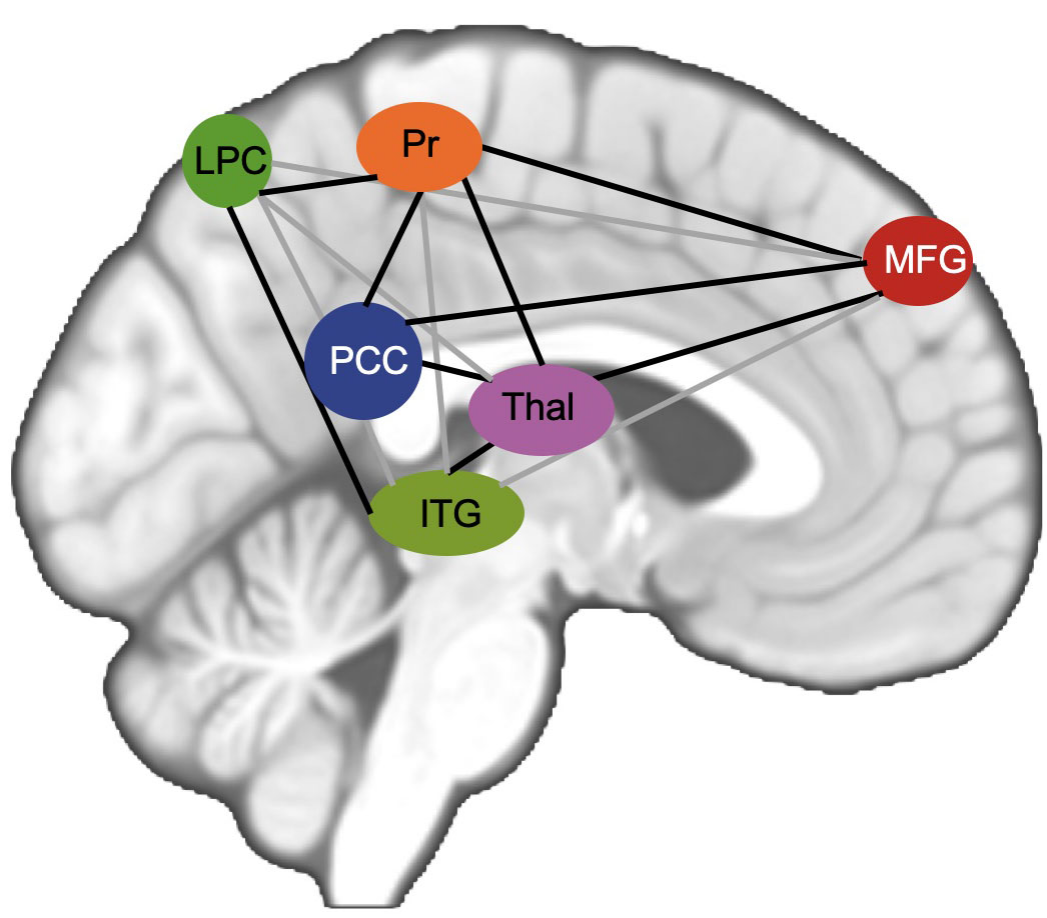
Figure shows schematic visualization of analyzed DMN. PCC posterior cingulate cortex in red, MFG middle frontal gyrus in green, Pr Precuneus, LPC lateral parietal cortex, ITG inferior temporal gyrus, Thal Thalamus.

In MDD patients, altered connectivity has been reported in a high number of regions of the brain, most prominently within the DMN, SN and EXE (23, 24, 28). Additionally, alterations in the cortico-limbic system, which plays a key role in mood regulation, have been reported in the past (26). To confine our results to interesting and canonical regions, we selected main seeds (71) for the networks mentioned above as our main seed for seed-based analysis to determine differences between MDD patients and HC (**Table 2**). Based on previous results concerning altered neural connectivity between HC and MDD (e.g. 26) and following the theory-guided deductive approach of Vega et al. (71), the posterior cingulate cortex (PCC) was used as region of interest (ROI) respectively seed for the DMN, the insula as seed for the SN (29), the hippocampus as seed for the cortico-limbic system (46) and the inferior parietal lobule (IPL) as seed for the EXE (45). Seed extraction was based on automatic anatomical labeling (AAL, 72). In order to confine our results between the selected main seed per network and voxels belonging to analyzed networks, pre-specified regions of interest (73) were analyzed, in order to reduce the severity of corrections for multiple tests. For every network that we examined, we selected a set of ROIs that has been recently reported as most commonly main parts of each analyzed rs-network. Following Li et al. (29) the LIM comprises ACC, amygdala and hippocampus, the DMN comprises the posterior cingulate cortex, precuneus, middle frontal gyrus, lateral parietal cortex, inferior temporal gyrus and the thalamus (**Figure 1**) and the executive control network contains the superior parietal lobule (SPL) along with ROIs of the frontal gyrus. In the SN the ACC (74) amygdala and the temporal pole as described by Mulders et al. (23) were included (**Table 2**, 60).

**Table 2:** Table shows ROIs of analyzed networks and the main hub belonging to each examined brain network.

Resting-state network	Main seed		MNI			Region of interest (AAL)		MNI		
			x	y	z			x	y	z
Cortico-limbic network (LIM)	Hippocampus	l	-26	-21	-10	Anterior cingulate cortex (ACC)	l	-5	35	14
		r	28	-20	-10		r	7	37	16
						Amygdala	l	-24	-1	-17
							r	26	1	-17
Executive control network (EXE)	Inferior parietal lobule (IPL)	l	-44	-46	47	Superior frontal gyrus	l	-19	35	42
		r	45	-46	50		r	21	31	44
						Middle frontal gyrus	l	-34	33	35
							r	36	33	34
						Inferior frontal gyrus, triangular	l	-47	30	14
							r	49	30	14
						Superior parietal lobule	l	-24	-60	59
							r	25	-59	62
Salience network (SN)	Insula (In)	l	-36	7	3	Anterior cingulate cortex (ACC)	l	-5	35	14
		r	38	6	2		r	7	37	16
						Amygdala	l	-24	-1	-17
							r	26	1	-17
						Temporal pole - Superior - Middle	l	-41 -37	15 15	-20 -34
							r	47 43	15 15	-17 -32
Default mode network (DMN)	Posterior cingulate cortex (PCC)	l	-6	-43	25	Precuneus	l	-8	-56	48
		r	6	-42	22		r	9	-56	44
						Middle frontal gyrus	l	-34	33	35
							r	37	33	34
						Lateral parietal cortex - Superior parietal lobule - Inferior parietal lobule	l	-24 -44	-60 -46	59 47
							r	25 45	-59 -46	62 49
						Inferior temporal gyrus	l	-51	-28	-23
							r	53	-31	-22
						Thalamus	l	-12	-18	8
							r	12	-18	8

Table shows names and abbreviations of examined seed-to-voxel analyzed AAL brain areas. Every examined resting-state network comprises a main seed and affiliated ROIs. For orientation and illustration purposes the MNI of the center of mass of each main seed is stated. The first letter in front of a region name indicates the left (l) or right (r) hemisphere of the brain.

### 2.8 Data analysis

Pearson’s correlation coefficients were computed between the time series of the aforementioned seeds and the time series of all other voxels in the brain to access FC. Correlation coefficients were then normalized using Fisher’s Z-transformation and compared between groups using two-sided t-tests. Results were considered significant when p < 0.05 using false discovery rate (FDR) correction for multiple comparisons on a cluster level. Bonferroni correction was applied to adjust for multiple comparisons. We performed 32 statistical tests simultaneously (16 tests for 2x2x2 ANOVAs and 16 tests for 2x2 ANOVAs). To adjust for an increased risk of alpha error, we performed Bonferroni correction as a multiple-comparison correction. The following p-values (**Tables 3**, **4**) remain significant after applying Bonferroni correction for 32 tests (tests (n) = 32, desired α = 0.05, α = 0.05/32 = 0.001).

**Table 3:** Table shows lower functional connectivity in MDD patients with ATT intervention compared to MDD patients who received sham intervention after one week of training.

seed	and	x	y	Z	p (FDR-corrected)	T	k
lPCC	lMFG	-32	38	36	0.030	4.76	153
	rMFG	32	40	40	0.030	4.56	62
rPCC	lMFG	-26	40	20	0.041	3.72	60

The letter in front of a region indicates the left (l) or right (r) hemisphere of the brain. PCC, posterior cingulate cortex; MFG, middle frontal gyrus.

**Table 4:** Table shows higher functional connectivity in HC who received ATT intervention compared to HC who received sham training after one week of training.

seed	and	x	y	z	p (FDR-corrected)	T	k
lPCC	lMFG	-36	14	58	0.005	4.53	142

The letter in front of a region indicates the left (l) or right (r) hemisphere of the brain. PCC, posterior cingulate cortex; MFG, middle frontal gyrus.

Using XJ View toolbox (https://www.alivelearn.net/xjview/) coordinates were localized based on automatic anatomical labeling (AAL). To locate the peak voxel of significant clusters, we used AAL (72).

### 2.9 Seed to voxel analysis

Seed to voxel analysis from baseline fMRI scan and from fMRI scan seven days later of the same individuals who have either received ATT intervention or sham intervention were evaluated performing mixed factorial ANOVA using SPM. As we aimed for a three-way interaction, a 2x2x2 ANOVA was performed with the between subject factors treatment (ATT vs sham) and state of illness (Healthy controls vs. MDD patients) and the within subject factor time (T0 vs T1) compelling 8 contrasts (16 contrast considering the left and right side of the brain). No significant differences in 2x2x2 ANOVA considering group, intervention and time were found. Then we compared MDD group and healthy controls separately using 2X2 ANOVA comprising the factors treatment (ATT vs sham intervention) and time (T0 vs T1). Primarily, we were interested in data in MDD patients after one week of ATT training. In order to make assumptions we also analyzed baseline differences and group differences involving the healthy control group after one week of ATT training.

## 3 Results

### 3.1 Demographics

48 individuals with MDD (30 women, 18 men; age 35.042+/- 11.15) and 51 HC (32 women, 19 mean age 33.725 +/- 7.77) were included in the study. Within the groups there were no significant differences between ATT and sham concerning gender, age, BDI (see **Table 1** for details).

### 3.2 BDI-II score

The 2x2x2 rmANOVA comprising the within-subject factor time (T0/T1) and the between-subject factors state of illness (MDD/HC) and treatment (ATT/sham) showed no significant interactions (time*treatment*state of illness, *F*(1, 98) = 0.476, *p* = 0.492, time*treatment: *F*(1, 98) = 0.501, *p* = 0.481 and time*state of illness: *F*(1, 98) = 3.321, *p* = 0.072).

### 3.3 Seed to voxel analysis

**Figure 2:**
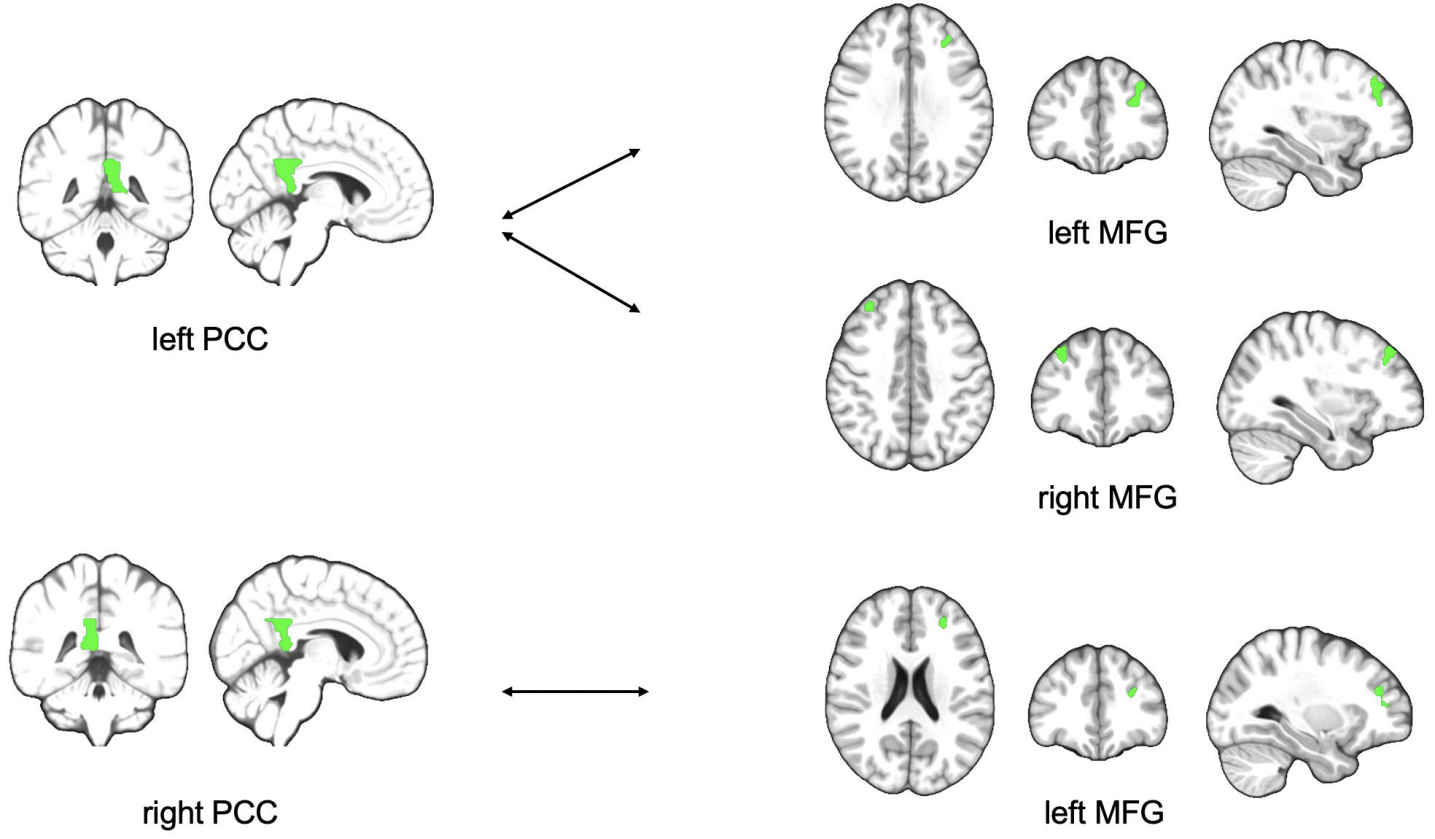
Figure shows schematic visualization of lower functional connectivity between PCC and MFG in MDD patients with ATT intervention compared to MDD patients who received sham intervention after one week of training PCC, posterior cingulate cortex; MFG, middle frontal gyrus.

**Figure 3:**
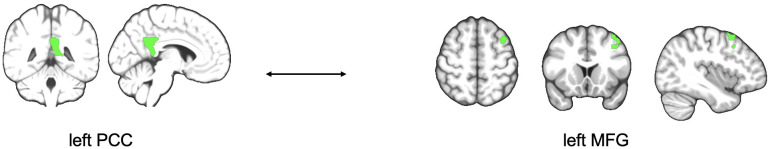
Figure shows schematic visualization of higher functional connectivity between left PCC and left MFG in HC who received ATT intervention compared to HC who received sham training after one week of training. PCC, posterior cingulate cortex; MFG, middle frontal gyrus.

There were no significant results in the established three factor model 2x2x2 ANOVA (time, treatment, state of illness). Nevertheless, a deeper look into the data reveals an interesting result analyzing 2x2 ANOVA. Comparing the subgroup of MDD patients only, the data shows lower functional connectivity in MDD patients obtaining ATT intervention compared to MDD patients who received sham intervention at the time of the second testing one week with daily intervention (T1) between left PCC and bilateral middle frontal gyrus (p=0.003 and p=0.041; **Table 3**) both ROIs belonging to the DMN. **Figure 1** shows the analyzed ROIs of the DMN. **Figure 2** shows a visual representation of the significant effect shown in **Table 3**. Moreover, for the subgroup of HC, data showed higher functional connectivity between left PCC and ipsilateral middle frontal gyrus in healthy controls who received ATT compared to healthy controls who received sham training after one week (p=0.005; **Table 4**). **Figure 3** shows a visual representation of the significant effect shown in **Table 4**. There were no significant results found in SN, LIM nor EXE.

## 4 Discussion

### 4.1 Discussion

There was no significant result in the three-factor model. It can be assumed that the present study suffers from a lack of statistical power for a 3-way interaction, as do many studies in neurology and psychiatry (75). Nevertheless, we decided to additionally look in the 2 groups separately for treatment effects. The results show a lower rsFC between left PCC and left MFG at the time of the second measurement in patients with MDD receiving ATT compared to the active control group of patients with MDD receiving sham. Furthermore, we found significantly higher rsFC between the same regions (left PCC and the left MFG) in HC who received ATT versus HC who received sham training intervention. Besides the PCC as the hub of the DMN, we could not find significantly altered rsFC between the hubs of the SN, EXE, or cortico-limbic systems and the associated regions of the corresponding neural network hubs. The combined tests of intervention, time and health status (MDD/HC) did not reach significance. The descriptive decrease in the BDI-II score in MDD who received ATT as a measure of the severity of the depressive episode did not reach significance.

Hyperconnectivity in the midline system of MDD, consisting of the PCC and medial frontal cortices, among others, has been found many times in studies with MDD and is considered a neural correlate of depressive symptoms based on the assumed functions of the areas, which also include rumination (e.g. 76–78). Frontal areas are thought to be involved in directing attention to self-relevant information (18) and the PCC is attributed to a role in the retrieval of autobiographical memories and self-focused thinking (77), among other activities. This midline system is active when self-relevant decisions are made (33). Therefore, higher rsFC of the associated areas (e.g. PCC and MFG) is often interpreted as a neural correlate of the rumination process, in which patients with MDD persist in negative, self-relevant thoughts (79, 80) and persistent negative preoccupation with the personal past, present and future (28). For example, in one fMRI study, activity in the ACC and the MFG has already been successfully used to differentiate healthy controls and patients with MDD (81). On a network level, the PCC and MFG are core hubs of the DMN. Differences in rsFC of the DMN have often been observed in association with MDD (37, 78, 82, 83). Commonly, patients with MDD showed higher rsFC (37, 51). In addition, DMN hyperconnectivity has been associated with an incapacity to stop rumination (48). Based on these results, we hypothesized that a psychotherapeutic treatment which is reported to successfully reduce the symptoms of rumination could be accompanied by a decrease in the functional hyperconnectivity of the DMN (10). For example, Hoch et al. (84) found a hint for the reduction of emotional dysregulation in MDD co-occurring with a reduced rsFC between the ACC and the MFG. In other words, decrease of the rsFC in the DMN occurred after treatment with cognitive-emotional training (Emotional Faces Memory Task). They argued the underlying mechanism of their results was due to short-term neural plasticity of the brain. Similar results for a reduction of rsFC in areas belonging to the DMN (PCC and MFG) in patients with MDD were found when patients were treated with mindfulness-based interventions (85), interventions to improve attention using meditation (86), and Emotion Regulation Therapy (19, 87). A similar pattern is also observed with the use of medication (82). DeMaster et al. (88) interpreted the increase in connectivity between ACC and PCC as the neurological basis of rumination in patients who were under medication.

Our findings suggest a decrease in rsFC between PCC and MFG in MDD when receiving ATT compared to sham. If we could also have shown that the performance in attention would be improved, then this would be in line with the proposed mode of action of ATT, as strengthening of the attention capacities should lead to a better detachment from self-centered thoughts (14). We present here the first study that followed the MCT treatment recommendation and applied a high-dose treatment with ATT. On a neural level, a reduction in rsFC between the PCC and the MFG in patients with MDD after the ATT intervention is observed. We note that changes in rsFC are possible in the group of MDD patients receiving a high dose of ATT. This may lead to further insight into the conceptual assumptions of ATT within MCT. However, this result at the neural level in association with reduced symptom severity or improved attention performance in patients with MDD would suggest neuronal mechanism that cause approximation of altered rsFC in MDD to rsFC in healthy controls. Normalization of dysfunctional connectivity between the PCC and MFG correspond to the concept of neuronal plasticity (89). Taken together, this result supports the idea that the process of maladaptive self-focus on negative aspects competes for resources of the brain’s processing capacity due to a lack of inhibition of the DMN, resulting in inhibition of outward-directed cognitive processes (18, 53). Following the conceptual assumptions of MCT, ATT serves to shift the focus of attention to less maladaptive external stimuli (11). When examining the brain using fMRI, a normalized pattern of connectivity of relevant network areas is then found in patients with depression receiving ATT. In the literature, a relatively consistent pattern of normalization of rsFC in the DMN is seen regardless of the therapeutic intervention (e.g. also in CBT; 90). According to the cognitive theory of depression (18), patients with MDD show systematic errors in the cognitive evaluation of experiences, which leads them to create and maintain a maladaptive view of themselves (91). These cognitive distortions in MDD patients combine increased attention to negative stimuli with increased attention to the self, known as negative self-focus (92). This self-centered attentional focus on subjectively negative aspects describes the process of rumination (81). Therefore, an improvement in the flexible voluntary shifting of attention stimulated by a psychotherapeutic intervention should enable the patient to ruminate less.

It should be noted that no significant reduction in depressive symptoms measured by the BDI-II was found after treatment with ATT compared to the sham condition. Only a significant decrease in the BDI-II score over time in both conditions (ATT and sham) was observed, indicating the possibility of a strong placebo effect in the sham condition. The reasons for the non-significant result are probably due to the design of the study. First, patients did not receive therapy according to the whole manual. Second, there was no psychoeducation or introduction to why ATT should be applied. ATT was studied as a standalone. Third, the sham condition also received an audio file with the same sounds as in the ATT audio file. The sham condition differed from the ATT condition only in the absence of instructions in the audio file. It is possible that the participants in the sham group also became active, for example due to boredom. It is possible that the construction of the sham condition led to sufficient placebo effects, which could complicate the interpretation of our results. Under these conditions, it is remarkable that there was still a significant decrease in symptomatology in ATT and sham conditions over time measured by the BDI-II.

In contrast to the patient group, we see an inverse effect in the healthy controls over time in the rsFC of the DMN based on ATT (i.e. decrease of rsFC in MDD and increase of rsFC in HC between PCC and MFG). To date, we have no valid hypothesis for this result. We need further investigation to explain the inverse findings in HC group. If this finding is replicated, this can be considered an initial indication of a new finding with clinical relevance.

### 4.2 Conclusion

The analysis showed no 2x2x2 effect. Nevertheless, we found an interesting result. This result cannot be interpreted as a treatment effect. The effect found suggests a change at the neural level, which should be investigated further. In line with the mct, one potential interpretation of the presented result is that changes in the rsFC in patients with MDD may reflect the clinical representation of depression, which is characterized by the persistence of the attentional focus on internal mental processes (28, 93). It has already been suggested in the literature that modifying the process of self-focus should be a central goal in psychotherapy (83). ATT strengthens attention, enabling patients to shift attention from internal processes to external ones (14). Our finding could tentatively suggest changes at the neural level related to high-dose application of ATT. We hypothesize that enhanced top-down control is achieved by intervening with ATT on network activity (94). Follow-up studies are needed to determine the quality of treatment prediction (95) and if the here identified network-based changes could be used for diagnostic purposes (30, 96). It would also be interesting for future research to investigate the neurotransmitter system after treatment with ATT (48), to include further clinical measurement data at the behavioral level and to perform further longitudinal studies (79). From a social perspective, knowledge about the neurobiological basis and effectiveness of psychotherapy can significantly strengthen the acceptance of psychotherapy in society and in the health sector and help to reduce the barriers to psychotherapeutic treatment for patients by providing a strong somatic understanding of illness (97).

### 4.3 Limitations

We only recruited patients with MDD who were not taking neuroleptics and had no comorbid diagnoses in alcohol and cannabis use disorders nor any psychotic disorders. Pre-selecting this group of patients with MDD on the one hand excluded possible confounding factors such as changes at the neural level caused by neuroleptics and drugs (78). Moreover, homogeneous samples, such as reported here, enable concrete and reliable conclusions (98). On the other hand, this often does not correspond to everyday clinical work (2, 27). This experiment focused on the external validity of the intervention through high-dose treatment with ATT. However, ATT was applied as a standalone treatment without embedding it in a therapeutic process through previous psychoeducation. This could result in an underestimation of the strength of the effect of ATT and explain the lack of significant change of depression symptoms in the sample.

Methodologically, a hypothesis-driven approach by focusing on one network node was adopted. Consequently, important information may have been excluded (79). Further focus could be given to whole-brain analysis of PCC and MFG connectivity independently in MDD and HC.

It is possible that the observed changes in the DMN are not causal but a consequence of changes in other connected networks (51). Statements on causal relationships cannot be made at this point, as cause and effect are still unclear.

Even though we knew that, according to theory, the DMN was the best candidate for our study, we planned to analyze 4 networks. We only found significant effects in the DMN. Moreover, we do not see an effect in the direct comparison between groups in the three-factor model but found effects only within the single groups (MDD or control group) caused by a leak in statistical power.

## Data Availability

The raw data supporting the conclusions of this article will be made available by the authors, without undue reservation.
